# Clinical outcomes of MR-guided adrenal stereotactic ablative radiotherapy with preferential sparing of organs at risk

**DOI:** 10.1016/j.ctro.2023.100680

**Published:** 2023-09-24

**Authors:** Famke L. Schneiders, Claire van Vliet, Nicolas Giraud, Anna M.E. Bruynzeel, Ben J. Slotman, Miguel A. Palacios, Suresh Senan

**Affiliations:** a Department of Radiation Oncology, Amsterdam-UMC, Location VUmc, The Netherlands; b Cancer Center Amsterdam, De Boelelaan 1117, 1081 HV Amsterdam, The Netherlands

**Keywords:** Adrenal metastases, SABR, MR-guided adrenal SABR, MRgRT, Underdosing, Coverage compromise index, Clinical outcomes

## Abstract

**Background and purpose:**

The optimal stereotactic ablative radiotherapy (SABR) doses for adrenal tumors are unknown. Some trials have specified that organ at risk (OAR) dose constraints should take priority over target coverage. We performed a retrospective review of the outcomes of MR-guided adrenal SABR (MRgRT) delivered with OAR sparing.

**Materials and methods:**

Patients who underwent adrenal MRgRT between 2016 and 2023 were identified from our Ethics-approved institutional database. Dose ranged between 8 and 24 Gy per fraction, delivered in 1–5 fractions. A 3 mm margin was added to the breath-hold gross tumor volume (GTV) to derive a PTV. Plan were delivered to an ‘optimized’ PTV that was generated by excluding any overlap with OARs.

**Results:**

Adrenal SABR was performed in 107 patients (114 metastases). The commonest scheme used 5 fractions of 10 Gy (53.5 %); 82 % of plans delivered a BED_10_ ≧ 80 Gy. Systemic therapy was administered within 3 months preceding or following SABR in 53.5 % of patients. Grade 3 acute toxicity (CTCAE v5.0) occurred in 0.9 % of patients, and 4.4 % reported late toxicity, consisting of adrenal insufficiency and a vertebral collapse. Median follow-up was 13.8 months (range, 0.0–73.4 months). Local progression occurred in 7.4 % of evaluable patients. PTV underdosage was frequent, with a coverage compromise index (D99/prescription dose) of < 0.90 in 52 % of all plans. Recurrences were independent of the prescription doses.

**Conclusion:**

MRgRT for adrenal metastases is well tolerated with high local control rates despite prioritizing OAR sparing over PTV coverage. Studies using deformable dose accumulation may lead to a better understanding of dose–response relationship with adaptive SABR.

## Highlights

• Daily adaptive adrenal SABR in ≤5 fractions is well tolerated, even in patients receiving systemic therapies.
• PTV underdosage was reflected by a coverage compromise index (D99/prescription dose) of <0.90 in 52% of all plans.
• In-field tumor progression was seen in 7.4% of patients, and independent of prescription dose.
• Deformable dose accumulation analysis may improve understanding of tumour dose-response relationships.

## Introduction

The treatment of selected patients presenting with a limited number of metastases using stereotactic ablative radiotherapy (SABR) has been incorporated in treatment guidelines in both North America and Europe [1]. While surgery is widely considered an effective treatment for adrenal metastases, incomplete resections have been reported in between 20 and 30 % of all patients [2], [3], [4]. Despite widespread adoption within the radiation oncology community, many aspects of patient selection and timing of SABR delivery remain unclear [5]. Metastases to the adrenal glands are a common manifestation of various tumors. While SABR has shown to be an effective and non-toxic treatment for adrenal lesions [6] there is currently no consensus on the optimal SABR doses required for achieving local control. A systematic review and pooled *meta*-analysis revealed that biological equivalent doses (BED_10_) of 60 Gy, 80 Gy and 100 Gy resulted in 1-year local control rates of 70.5 %, 84.8 %, and 92.9 % [6]. However, some ongoing trials like SABR-COMET 10 require use of single-fractions (SF) of 20 Gy, or 3 fractions of 10 Gy (both BED = 60), to ≧ 4 metastases in order to minimize potential risks associated with treating multiple sites [7]. These and other trials have specified that organ at risk (OAR) dose constraints should take priority over target coverage in treatment planning, permitting PTV underdosage if needed.

The recent introduction of MR-guided SABR (MRgRT) has allowed for better on-couch imaging of both adrenal tumors and OARs, in comparison to online kV-CBCT imaging, which in turn facilitates daily adaptive re-planning to improve OAR sparing and target coverage [8]. However, a preference for OAR sparing could potentially compromise local control with MRgRT, and the available literature on MRgRT is limited to small patient series with short follow-up. The current study has two main aims; firstly, to study the toxicity of adrenal SABR delivered using an OAR-sparing, gated breath-hold MRgRT approach in patients undergoing treatment with contemporary systemic agents, and secondly, assess long-term local control rates in patients who undergo daily gated breath-hold MRgRT with on-couch adaptive and prevalence of OAR sparing.

## Methods

Patients who underwent MRgRT SABR for adrenal metastases between 2016 and 2023 were identified from our institutional Ethics-approved database. This retrospective study was granted exemption by our Medical Ethics Review Committee. Our MRgRT adrenal SABR procedure on a MRIDIAN unit (ViewRay, Mountain View) during repeated breath-holds has been reported in detail previously [8]. Briefly, an MR scan is repeated before each fraction, followed by rigid coregistration to the gross tumor volume (GTV) on the pretreatment MR scan. Contour deformation, planning target volume (PTV) (GTV + 3 mm) expansion, and online plan reoptimization were then performed. A 3D MR simulation was acquired during inspiration breath-hold for pretreatment delineation and generation of a treatment plan, and 3D MR acquisitions were also performed prior to each daily fraction. Online new OAR contours were generated by Deformable Image Registration (DIR) and adjusted at the discretion of the physician within 2 cm of planned tumor volume (PTV) on the daily MR, and the online plan was optimized. The entire adrenal gland was contoured in the GTV even when the tumor deposit was localized in just one region of the adrenal on imaging studies, including FDG-PET scans. The PTV was derived by adding a 3 mm margin to the breath-hold gross tumor volume (GTV), and an ‘optimized’ PTV was generated by excluding the overlap with the OARs. Reoptimized plans were generated each day, which was always found dosimetrically superior to the original plan. The contoured GTV was tracked on the feedback monitor, and visible gating boundary of 3 mm minus overlapping OARs was added to the GTV. A step and shoot IMRT technique was delivered in 1–8 fractions, with beam-on only when the target is inside the PTV, which was used as gating boundary during delivery. During delivery, visual feedback by means of an MR-compatible monitor displaying the cine-MR with the target and PTV structures was used to assisted the patient with breath-hold reproducibility. Patients were initially treated using mainly a 5-fraction scheme, but 3-fraction and single fraction schemes were adopted from 2020 [6]. The protocol specified a maximum hot spot of 125–140 % of the prescribed dose, if possible. Institutional OAR constraints to the stomach, bowel, and duodenum for the 5 fractions of 10 Gy scheme were V36Gy, V33Gy, and V25Gy, and should ideally be lower than 0.1, 1.0, and 5 cm3, respectively [Palacios et al IJROBP 2018]. Patients were instructed to fast for approximately 2.5 h before simulation. Anti-emetics were prescribed only when indicated. Patient characteristics and treatment details were collected, and outcomes were reported per treated adrenal metastasis. Oligometastatic state was defined as per ESTRO-ASTRO consensus definitions [1]. Follow-up details and radiological investigations were obtained from referring hospitals and general practitioners. Toxicity from stereotactic MR-guided adaptive radiotherapy (SMART) was measured using the CTCAE v5.0. Baseline tumor sizes were measured on the planning-CT scan. Post-SABR responses were measured in the axial plane on the evaluation CT scans using RECIST v1.1 criteria, and reviewed by a second radiation oncologist. The GTV, PTV and optimized planned tumor volume (excluding overlap with OARs from the PTV) were recorded. Overall (OS), progression-free (PFS) and distant recurrence-free survivals (DRFS) were calculated from the date of the last fraction of radiotherapy. Various patient, tumor and treatment characteristics were analyzed as prognostic factors for local control, PFS and OS, including age, sex, primary histopathology, BED, initial cancer stage, metastatic timing and pattern, use of single fraction, GTV and PTV sizes, coverage compromise index (CCI, defined as D99/prescription dose), concurrent chemotherapy or immunotherapy. All analyses were performed using R (v4.2.2) and RStudio (v2022.12.0 + 353) software. Descriptive statistics were used to describe basic-, tumor-, and treatment characteristics. Survival analyses were performed using the Kaplan-Meier estimate. Local responses were assessed for each treated adrenal metastasis separately. In patients treated sequentially for a second adrenal metastasis, only the first SABR delivery was considered for determining the PFS and OS. Univariate Cox proportional hazard models were used to assess the potential influence of patient, tumor, and treatment characteristics on local control, PFS and OS, associated with the respective hazard ratios (HR) and their 95 % confidence interval (95 %CI). A p-value < 0.05 was deemed statistically significant.

## Results

Between 2016 and 2023, a total of 107 patients underwent SABR using MRgRT to 114 adrenal metastases. Patient and tumor characteristics are summarized in Table 1. The main primary tumors were non-small cell lung cancer (67.3 %) and renal cancer (7.5 %). The median interval from diagnosis of the primary cancer to adrenal SABR was 22.9 months (range, 1.5–156.9 months). Seven patients underwent bilateral adrenal SABR, of which 3 presented with synchronous lesions and 4 with metachronous lesions, at intervals ranging from 11 to 31 months. The largest patient subgroup comprised those with either solitary adrenal metastasis (40.4 %) or with 2–5 metastatic lesions (53.5 %). The median tumor diameter was 34 mm (range, 13–90 mm), and median GTV was 24.4 cc (range, 1.3–252.1 cc). The commonest fractionation scheme used was 5 fractions of 10 Gy (53.5 %), and most plans delivered a BED_10_ ≧ 80 Gy (81.6 %)(Table 2). Single fraction SABR using doses ranging from 16 to 24 Gy was delivered in 13.2 % of patients.

**Table 1:** Patient and tumor baseline characteristics.

Patient characteristics (*n* = 107)	
Sex	
Female	34 (31.8 %)
Male	73 (68.2 %)
Age (y), median (range)	66 (41–90)
Performance score (n = 105)	
WHO 0	28 (26.7 %)
WHO 1	55 (52.4 %)
WHO 2	20 (19.0 %)
WHO 3	2 (1.9 %)
Primary tumor	
Lung non-small cell	72 (67.3 %)
Renal cell	8 (7.5 %)
Colorectal	5 (4.7 %)
Hepatocellular	5 (4.7 %)
Lung small-cell	4 (3.7 %)
Melanoma	4 (3.7 %)
Esophageal	2 (1.9 %)
Other	7 (6.5 %)
Tumor stage at initial diagnosis	
Stage I	13 (12.1 %)
Stage II	10 (9.3 %)
Stage III	33 (30.9 %)
Stage IV	51 (47.7 %)
Tumor characteristics (*n* = 114)	
Side	
Left	65 (57.0 %)
Right	49 (43.0 %)
Max diameter (mm), median (range)	34 (13–90)
Primary tumor controlled	
Yes	94 (82.5 %)
No	20 (17.5 %)
Biopsy proven metastasis	
Yes	43 (37.7 %)
No	71 (62.3 %)
PET staging performed prior to SABR	
Yes	90 (78.9 %)
No	24(21.1 %)
Presentation of adrenal metastasis (as per ESTRO-ASTRO consensus definitions)	
Synchronous	16 (14.0 %)
Metachrone oligorecurrence	44 (38.6 %)
Oligoprogression	53 (46.5 %)
Oligopersistance	1 (0.9 %)
Metastatic Pattern	
Solitary	46 (40.4 %)
Other Oligometastases (2–5)	61 (53.5 %)
Multiple	7 (6.1 %)
Systemic therapy	
Within 3 months	59 (51.8 %)
During/concurrent	22 (19.3 %)
After (>3 mo)	33 (28.9 %)

**Table 2:** SABR and planning details.

*n* = 114	
Dose/fractionation	
5x 10 Gy	61 (53.5 %)
3x 15 Gy	15 (13.2 %)
1x 24 Gy	12 (10.5 %)
5x 8 Gy	11 (9.6 %)
4x 10 Gy	4 (3.5 %)
Other*	11 (9.7 %)
Single fraction 16–24 Gy	15 (13.2 %)
BED_10_ ≥ 80 Gy	93 (81.6 %)
BED_10_ ≥ 100 Gy	77 (67.5 %)
GTV volume (cc), median (range)	24.4 (1.3–252.1)
PTV volume (cc), median (range)	40.2 (3.6–319.2)
Treatment finished	111 (97.4 %)
Prescription dose, mean (95 % CI), Gy	43.4 (41.6–45.3)
No. of fractions, mean (95 % CI)	4.1 (3.8–4.4)
Prescription isodose line, mean (95 % CI)	79.6 (78.7–80.4)
Dmax (Gy), mean (95 % CI)	54.7 (52.4–57.1)
D1 (Gy), mean (95 % CI)	53.3 (51.0–55.6)
D95 (Gy), mean 95 % CI)	41.2 (39.4–43.0)
D99 (Gy), mean 95 % CI)	34.7 (32.8–36.6)
CCI, mean (95 % CI)	0.81 (0.78–0.84)
CCI > 0.90, n (%)	54 (48 %)
CCI > 0.96, n (%)	16 (14 %)

Abbreviation: CCI = coverage compromise index, defined as D99/prescription dose. A CCI value < 0.90 indicates coverage compromise, as the SABR-COMET protocol indicated that 99 % of the PTV was to be covered by 90 % of the prescription dose.

*1*16 Gy (n = 1), 1*20 Gy (n = 2), 3*8Gy (n = 2), 3*10 (n = 4), 5*7 Gy (n = 1), 8*7.5 Gy (n = 1).

Planned fractions were completed in 97.4 % of cases. Grade 3 acute toxicity (CTCAE v5.0) was seen in 0.9 % and in 4.4 % of patients late toxicity was reported (adrenal insufficiency and vertebral collapse) (Table 3). A patient known to have anticipatory nausea to chemotherapy did not complete planned SABR after 3 fractions due to grade 3 nausea with weight loss, and another due to out-of-field disease progression. Treatment in another patient was interrupted after a single fraction of 15 Gy due to spontaneous adrenal hemorrhage, and SABR was later resumed to deliver 4 additional fractions of 10 Gy. Late toxicity was seen in 5 patients; 3 patients developed a collapse of the first lumbar vertebra, and another 2 developed grade 2 adrenal insufficiency (one of whom was treated synchronously on bilateral adrenals). One vertebral fracture was diagnosed at 5 months post-SABR [plan parameters: D0.1 cc 40.11 Gy, D1% 36.15 Gy, D98% 5.49 Gy] and was treated with vertebroplasty (grade 3). A second patient developed a grade 1 vertebral fracture at 6 months post-SABR [D0.1 cc 30.23 Gy, D1% 25.92 Gy, D98% 6.77 Gy], and a third developed a grade 1 vertebral collapse at 8 months post-SABR [D0.1 cc 41.98.2 Gy, D1% 36.48 Gy, D98% 4.62 Gy].

**Table 3:** Toxicity scored using CTCAE v5.0.

*n* = 114	
Acute toxicity grade 1–2	65 (57.0 %), 8 (7.0 %) grade 2
Fatigue	42 (36.8 %), 3 (2.6 %) grade 2
Nausea	23 (20.2 %), 5 (4.4 %) grade 2
Diarrhea	8 (7.0 %), 1 (0.9 %) grade 2
Anorexia	2 (1.8 %), 1 (0.9 %) grade 2
Abdominal pain	2 (1.8 %), 0 grade 2
Other	2 (1.8 %), 0 grade 2
Acute toxicity grade ≥ 3	1 (0.9 %)
Nausea	1 (0.9 %)
Late toxicity all grade	5 (4.4 %), 1 (0.9 %) grade ≥ 3
Adrenal insufficiency	2 (1.8 %), 2 grade 2
Vertebral collapse	3 (2.6 %), 1 (0.9 %) grade 3

**Fig. 1:**
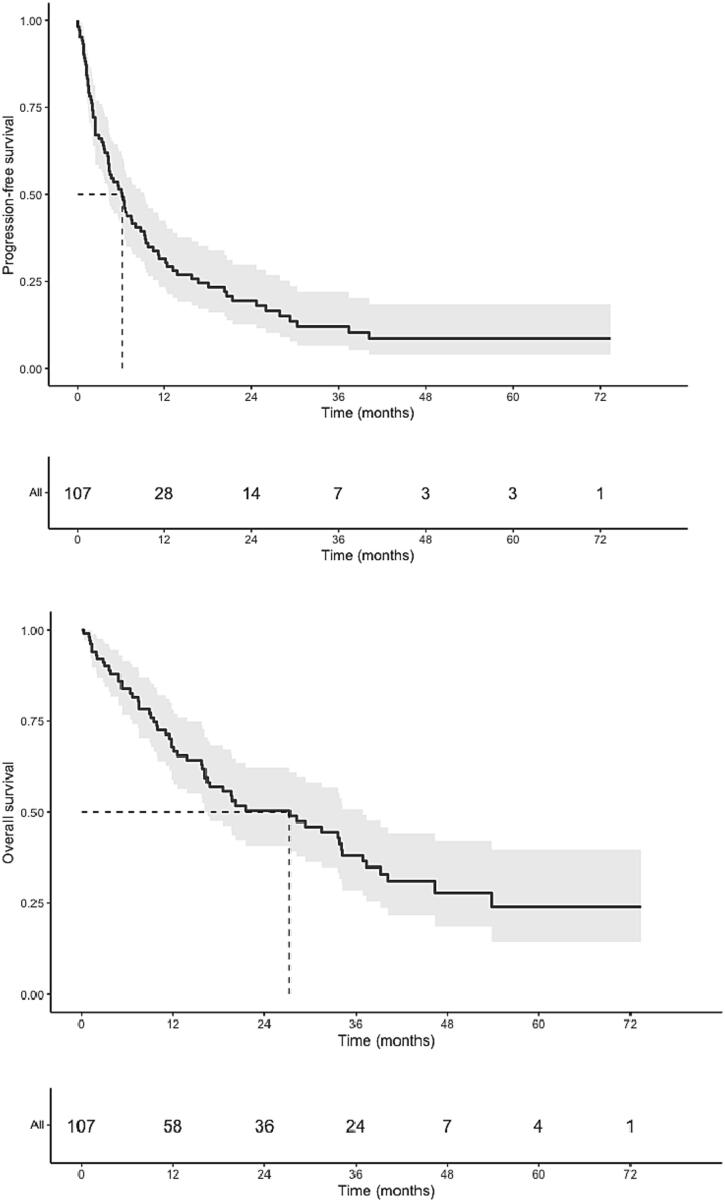

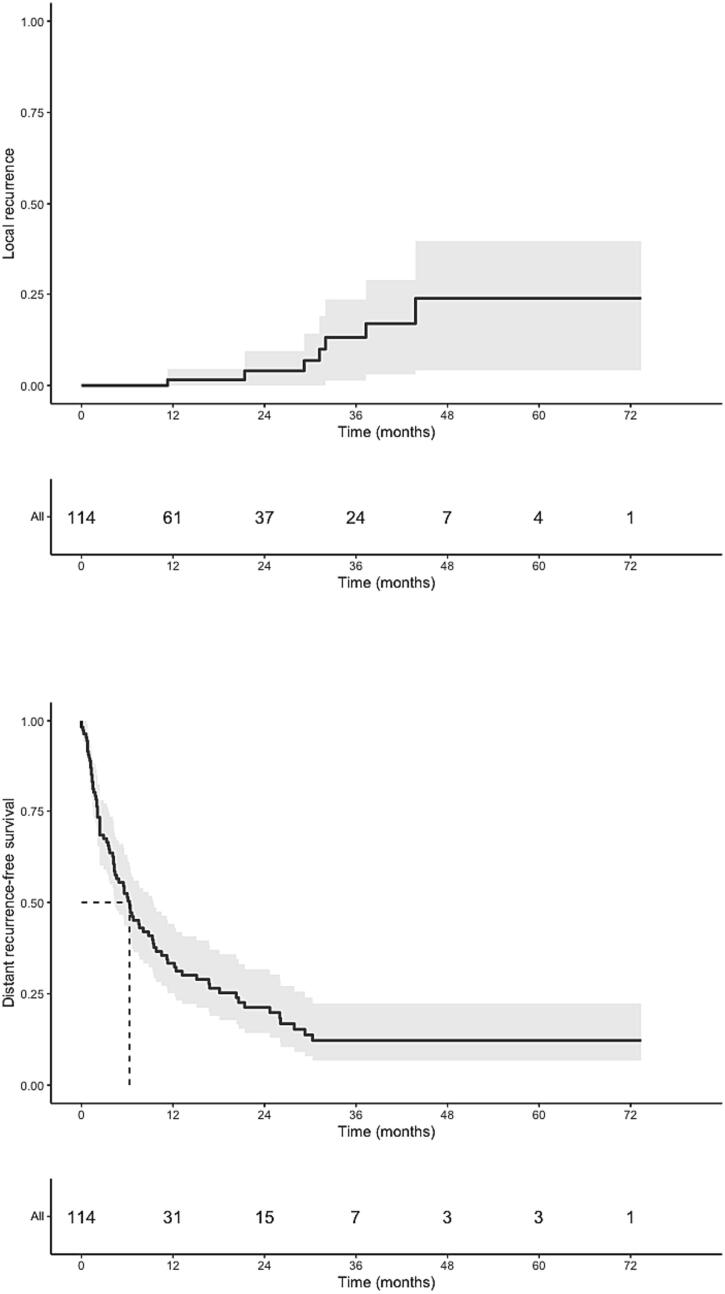
PFS (A), OS (B), LR (C) and DRFS (D).

Within a timeframe of 3 months preceding or following SABR, 53.5 % of all patients received chemotherapy, immunotherapy or targeted therapy (Table 4). Of these, 19.3 % of patients received some form of systemic therapy concurrently with SABR. Median follow-up was 13.8 months (range, 0.0–73.4 months), and the best RECIST local responses in 95 evaluable patients were stable disease (SD, 30.5 %), partial response (PR, 40.0 %) and a complete response (CR, 22.1 %), respectively. Local progression was observed in 7.4 % of evaluable patients (6.1 % of the overall cohort); the median interval to local progression was 31.2 months (range, 11.3–43.8 months). No isolated recurrences were observed. In total, 72.0 % patients had out-of-field progression, with most (83.1 %) manifesting within one year of SABR (Fig. 1). OS and PFS rates were respectively 67.8 % (95 %CI 58.9–78.1) and 31.5 % (95 %CI: 23.4–42.56) at one year; 50.4 % (95 %CI 40.8–62.2) and 19.5 % (95 %CI: 12.7–29.8) at two years. Median OS was 27.3 months (95 %CI 16.2–36.9), and median PFS was 6.1 months (95 %CI: 4.2–9.3) (Table 5).

**Table 4:** Details of systemic therapy details delivered within a 3-month window of receiving adrenal MRgRT.

*n* = 114	
Chemotherapy	21 (18.4 %)
Immunotherapy	33 (28.9 %)
Targeted therapy	7 (6.1 %)
Continued during MRgRT	22 (19.3 %)
Type of therapy within 3 months	
Pembrolizumab	18 (15.8 %)
Carbo/Cisplatin	15 (13.2 %)
Pemetrexed	10 (8.8 %)
Nivolumab	10 (8.8 %)
Durvalumab	6 (5.3 %)
Encorafenib	2 (1.8 %)
Afatinib	1 (0.9 %)
Osimertinib	1 (0.9 %)
Gefitinib	1 (0.9 %)
Trastuzumab	1 (0.9 %)
Type of therapy continued during MRgRT	
Pemetrexed	1
Pembrolizumab	9
Nivolumab	4
Durvalumab	2
Atezolizumab	1
Afatinib	1
Trastuzumab	1
Gefitinib	1
Encorafenib	1
Osimertinib	1

**Table 5:** Clinical outcomes.

Median follow-up (months, range)	13.8 (0.0–73.4)
Adrenal response (available for *n* = 95)	
Stable	29 (30.5 %)
Partial	38 (40.0 %)
Complete	21 (22.1 %)
Progression	7 (7.4 %)
1-year Local control	98.5 % (95.5–100)
2-year Local control	96.0 % (90.6–100)
1-year Distant recurrence-free survival	33.4 % (25.2–44.2)
2-year DRFS	21.3 % (14.3–31.7)
Median DRFS (months, 95 %CI)	6.3 (4.3–9.4)
1-year OS (95 %CI)	67.8 % (58.9–78.1)
2-year OS (95 %CI)	50.4 % (40.8–62.2)
Median OS (mo, 95 %CI)	27.3 (16.2–36.9)
1-year PFS (95 %CI)	31.5 % (23.4–42.5)
2-year PFS (95 %CI)	19.5 % (12.7–29.8)
Median PFS (mo, 95 %CI)	6.1 (4.2–9.3)

A borderline significant relationship was seen between CCI < 0.90 and local control in the univariate analyses (HR 0.11p = 0.049, 95 %CI: 0.01–0.99). No significant correlations were observed for local control and the BED_10_ (<or ≥ 100 Gy, p = 0.29, HR 0.44 (95 %CI: 0.10–1.99)) or GTV size (p = 0.28, HR 1.01 (95 %CI: 0.99–1.03)). In multivariable analyses, when adding other variables such as GTV size or BED_10_, CCI < 0.90 did not remain significant.

A worse PFS correlated with larger GTV size (p = 0.037, HR 1.004 (95 %CI: 1.00–1.01) and receipt of concurrent chemotherapy ± 3 months (p = 0.002, HR 2.28 (95 %CI: 1.35–3.84)). Receipt of concurrent immunotherapy ± 3 months was associated with improved PFS (p = 0.033, HR 0.58 (95 %CI: 0.36–0.96). The PFS did not correlate with dose in BED_10_ (p = 0.64, HR 1.003 (95 %CI: 0.99–1.02)). For OS, only concurrent chemotherapy ± 3 months (p = 0.01, HR 2.07 (95 %CI: 1.17–3.65)) was associated with poorer outcomes.

Characteristics of patients with a follow-up exceeding 12 months, and without out-of-field progression (*n =* 15) are summarized in Table S1 (Supplementary Data). These patients had mainly a diagnosis of NSCLC (66.7 %), oligorecurrence (20.0 %) or oligoprogressive (60.0 %) disease. Importantly, 66.7 % of all such patients had attained a CR, as compared to 23.1 % of evaluable patients with > 3 months follow-up.

## Discussion

Our single institution study of daily adaptive adrenal MRgRT using breath-hold delivery revealed a 1-year local control of 98.5 % (CI 95.5–100) and a 2-year local control of 96.0 % (CI 90.6–100). No excessive toxicity was observed even though more than 80 % of patients had a SABR plan delivering a BED_10_ ≧ 80 Gy, and the receipt of contemporary systemic therapy in 53.5 % of patients. To the best of our knowledge, this is the largest reported experience to date with adrenal MRgRT, and adds to the growing body of literature showing that SABR is a safe and effective treatment of adrenal metastases. The low toxicity despite use of immunotherapy is consistent with a pooled patient-level analysis of adverse events after this treatment, either with or without radiotherapy [9]. We observed a 0.9 % grade 3 acute toxicity (CTCAE v5.0) and 4.4 % late toxicity (adrenal insufficiency and vertebral collapse). The significance of a vertebral collapse in 3 patients is unclear given the age of our treated population, and this finding has been previously reported after thoracic chemoradiotherapy with relatively low radiation doses and at a short interval following radiotherapy [10]. The low toxicity and high control rates reported in our patient population contrast with, and highlight the advantages of SABR over surgery, where rates of incomplete resections are approximately 20–30 % [2], [3], [4], and when risk of interruptions to systemic therapy must be minimized.

The CCI has been used to measure the trade-off between OAR doses and PTV coverage in the SABR-COMET and SABR 5 trials. Our adrenal MRgRT plans had a CCI exceeding 0.90 in 48 % of all cases, and it was > 0.96 in just 14 % of treated patients. Despite this, we did not observed differences in dose-related local control rates for either using a BED_10_ 80 or 100 Gy. The SABR-COMET trial protocol specified CCI values < 0.9 as indicative of compromised target coverage, and this was noted in all 7 adrenal metastases treated [11]. Despite this, lower CCI values were not related to adverse consequences on the OS, PFS or local control in this study [11]. Similarly, the larger phase II SABR-5 study also reported that compromised target coverage did not predict for significantly worse local control or PFS [12]. These findings suggest that high SABR doses to the whole PTV may not be essential for OMD treated with modern radiotherapy techniques and appropriate in-room imaging. In contrast, earlier reports had suggested that delivery of a BED_10_ ≧70 Gy was necessary for adrenal metastases [6], [13], [14]. The use of lower BED schemes for OMD for less favorable patients’ subgroups, such as for those eligible for SABR-COMET 10, appears justified [7]. Although our univariate analysis indicated that CCI < 0.90 was predictive for local relapse, other confounding factors should be acknowledged, such as a greater likelihood of compromised target coverage for larger tumors, and our use of only baseline SABR plans for our analysis, thereby not taking daily adaptation into account.

Use of MR-guidance for adrenal SABR allowed for the delivery of ablative doses in 5 or fewer fractions with better OAR sparing. The use of 1- and 3-fraction SABR are more patient-friendly and resource efficient, and these schemes are generally not performed on our CT-linacs, where adaptive techniques are not used. Our MRgRT fractionation schemes are similar to those recommended in primary renal cell cancer, namely one, three or five fraction SABR to 25–26 Gy, 35–45 Gy, and 40–50 Gy, respectively [15]. The best RECIST local responses in 95 evaluable patients were SD (30.5 %), PR (40.0 %) and a CR (22.1 %). In contrast, RECIST responses after renal SABR have reported a SD in 75 %, a PR in 19 % and a CR in just 2.4 % [16]. Our MRgRT dose fractionation schemes are determined by the proximity of critical OARs [17], and the current results do not indicate the superiority of any one of the schedules. More data is needed, including from ongoing studies that are evaluating potential immunomodulatory effects of SABR schedules [18].

Our findings must be considered in the light of the strengths and limitations of this analysis. We analyzed MRgRT outcomes at a single institution, where patients were rapidly accrued over a 6-year period and treated using a uniform OAR-sparing approach. Limitations include the retrospective nature of the study, and the heterogeneity of the patient cohort regarding histology of primary tumors and extent of disease, with 40 % presenting with solitary adrenal metastases and most treated for oligoprogressive disease (60 %). Furthermore, there are variations in systemic treatment regimens used in the 53.5 % of patients who received systemic therapies within 3 months’ time, and in the 19 % of patients receiving concurrent systemic treatments. Patient follow-up was limited to a median in the overall population of just 13.8 months, whereas median interval to local progression in our series was 31.2 months. In addition, the current analyses were performed using the baseline dose plan data, and future analyses using deformable dose accumulation may reveal additional insights.

In conclusion, the toxicities and outcomes of MRgRT for adrenal metastases are favorable despite placing a higher priority on OAR sparing over PTV coverage. Longer follow-up and studies using deformable dose accumulation may lead to a better understanding of the role of SABR underdosing for this disease site.

## Conflicts of interest statement

The Department of Radiation Oncology at the Amsterdam University Medical Center has funded research agreements with ViewRay Inc. and Varian Medical Systems.

## Financial support

No industry funding was received for the planning and conduct of this study. The content of the work is solely the responsibility of the authors.

## CRediT authorship contribution statement

**Famke L. Schneiders:** Conceptualization, Resources, Formal analysis, Writing – original draft, Project administration, Investigation, Validation. **Claire van Vliet:** Writing – review & editing, Investigation, Validation. **Nicolas Giraud:** Resources, Writing – review & editing, Data curation, Formal analysis, Visualization. **Anna M.E. Bruynzeel:** Resources, Writing – review & editing. **Ben J. Slotman:** Resources, Writing – review & editing. **Miguel A. Palacios:** Resources, Writing – review & editing. **Suresh Senan:** Conceptualization, Resources, Writing – review & editing, Validation, Supervision.

## Declaration of Competing Interest

The authors declare that they have no known competing financial interests or personal relationships that could have appeared to influence the work reported in this paper.
