# Supplementary material for: Clinical outcomes of MR-guided adrenal stereotactic ablative radiotherapy with preferential sparing of organs at risk
Source: Clin Transl Radiat Oncol. 2023 Sep 24;43:100680. doi: 10.1016/j.ctro.2023.100680 (PMC10551830; doi:10.1016/j.ctro.2023.100680)
Supplement: Supplementary data 1 [file mmc1.docx]

**Supplementary data**

**Table S1.** Characteristics of patients followed-up for >12 months without progressive disease

*n*=15

Age (y), median (range) 66 (47-81)

Histology
 Lung NSCLC 10 (66.5%)
 Renal carcinoma 1 (6.7%)
 Hepatocellular 1 (6.7%)
 Neuroendocrine 1 (6.7%)
 Melanoma 1 (6.7%)

Endometrial carcinoma 1 (6.7%)
Oligocategory (as per ESTRO-ASTRO consensus definitions)
 Synchronous 3 (20.0%)
 Meta-oligorecurrence 3 (20.0%)
 Repeat oligoprogression 9 (60.0%)
RT dose schedule
 5x 10 Gy 7 (46.8%)
 3x 15 Gy 2 (13.3%)
 1x 24 Gy 2 (13.3%)
 3x 8 Gy 2 (13.3%)
 5x 8 Gy 2 (13.3%)
BED >100 9 (60.0%)
BED >80 15 (100.0%)

D1 (Gy), mean (95% CI) 44.4 (35.8-52.9)

D95 (Gy), mean 95% CI) 35.4 (28.8-42.0)

D99 (Gy), mean 95% CI) 31.4 (25.4-37.5)

CCI, mean (95% CI) 0.88 (0.82-0.94)

CCI>0.90, n (%) 11 (73%)

CCI>0.96, n (%) 2 (13%)

IO within 3 months 8 (53.3%)
Local control
 Complete response 10 (66.7%)
 Partial response 4 (26.7%)

Stable 1 (6.6%)
